# The ecology and bioactivity of some Greco-Roman medicinal minerals: the case of Melos earth pigments

**DOI:** 10.1007/s12520-021-01396-z

**Published:** 2021-09-17

**Authors:** C. W. Knapp, G. E. Christidis, D. Venieri, I. Gounaki, J. Gibney-Vamvakari, M. Stillings, E. Photos-Jones

**Affiliations:** 1 Civil and Environmental Engineering, Strathclyde University, Glasgow, UK; 2 Mineral Resources Engineering, Technical University of Crete, Chania, Greece; 3 Environmental Engineering, Technical University of Crete, Chania, Greece; 4 Adamas, Milos Greece; 5 Archaeology, School of Humanities, University of Glasgow, Glasgow, UK; 6 Analytical Services for Art and Archaeology, Ltd, Glasgow, UK

**Keywords:** Ochres, Earth pigments, Jarosite, Melanterite, Manganese oxide, *Miltos*, Ecology, Antibacterial activity

## Abstract

Mineral compounds, as pigments and therapeutics, appeared regularly in the technical and medical texts of the Greco-Roman (G-R) world. We have referred to them as ‘G-R medicinal minerals’ and we suggest that despite their seeming familiarity, there are actually many unknowns regarding their precise nature and/or purported pharmacological attributes. Earth pigments are part of that group. This paper presents a brief overview of our work over the past twenty years relating to: a. the attempt to locate a select number of them in the places of their origin; b. their chemical/mineralogical characterization; c. the study of their ecology via the identification of the microorganisms surrounding them; d. their testing as antibacterials against known pathogens. In the process, and to fulfil the above, we have developed a novel methodological approach which includes a range of analytical techniques used across many disciplines (mineralogy, geochemistry, DNA extraction and microbiology). This paper focuses on a select number of earth pigments deriving from the island of Melos in the SW Aegean, celebrated in antiquity for its *Melian Earth*, a white pigment, and asks whether they might display antibacterial activity. We demonstrate that some (but not all) yellow, green and black earth pigments do. We also show that the manner in which they were dispensed (as powders or leachates) was equally important. The results, although preliminary, are informative. Given their use since deep time, earth pigments have never lost their relevance. We suggest that the study of their ecology/mineralogy and potential bioactivity allows for a better understanding of how our perception of them, as both pigments and therapeutics, may have evolved.

## PREMISE

This Topical Collection (TC) covers several topics in the field of study, in which ancient architecture, art history, archaeology and material analyses intersect. The chosen perspective is that of a multidisciplinary scenario, capable of combining, integrating and solving the research issues raised by the study of mortars, plasters and pigments (Gliozzo et al. 2021).

The first group of contributions explains how mortars have been made and used through the ages (Arizzi and Cultrone 2021, Ergenç et al. 2021, Lancaster 2021, Vitti 2021). An insight into their production, transport and on-site organisation is further provided by DeLaine (2021). Furthermore, several issues concerning the degradation and conservation of mortars and plasters are addressed from practical and technical standpoints (La Russa and Ruffolo 2021, Caroselli et al. 2021).

The second group of contributions is focused on pigments, starting from a philological essay on terminology (Becker 2021). Three archaeological reviews on prehistoric (Domingo Sanz and Chieli 2021), Roman (Salvadori and Sbrolli 2021) and Medieval (Murat 2021) wall paintings clarify the archaeological and historical/cultural framework. A series of archaeometric reviews illustrate the state of the art of the studies carried out on Fe-based red, yellow and brown ochres (Mastrotheodoros et al. 2021), Cu-based greens and blues (Švarcová et al. 2021), As-based yellows and reds (Gliozzo and Burgio 2021), Pb-based whites, reds, yellows and oranges (Gliozzo and Ionescu 2021), Hg-based red and white (Gliozzo 2021) and organic pigments (Aceto 2021). An overview of the use of inks, pigments and dyes in manuscripts, their scientific examination and analysis protocol (Burgio 2021) as well as an overview of ​glass-based pigments (Cavallo and Riccardi 2021) are also presented. Furthermore, two papers on cosmetic (Pérez Arantegui 2021) and bioactive (antibacterial) pigments (this paper) provide insights into the variety and different uses of these materials.

## Introduction

### The pharmacology of G-R medicinal minerals: A methodological approach for the examination of biome-rich minerals

‘Greco-Roman (G-R) medicinal minerals’ (G-R MMs) is a term we have put in place to refer to the minerals/minerals combinations that feature in pharmacological preparations in the medical and technical texts of antiquity: Theophrastus (*On Stones*), Pliny (*Natural History*, Book 35), Dioscorides (*De Materia Medica*), Celsus (*De Medicina*), Galen (many texts), Scribonius Largus (*Compositiones*), and in numerous commentaries of them throughout the medieval, Renaissance and modern periods. Although most of the c. 90 minerals (minerals combinations and rocks) grouped together in Dioscorides’ Book 5 (Beck 2005), appear familiar to the reader, they are actually little understood from the perspective of both their nature (mineralogical/geochemical) and their pharmacological attributes.

There are many reasons for that: some G-R MMs have known geographical places of origin (for example, *Lemnian Earth *(*Mat. Med*. V.97) or *Melian* *Earth* (*Mat. Med*. V.159) but their localities of extraction are not specified, making their identification in the field more laborious; for others, the region is too broad or vaguely defined to be of practical use (*chrysocolla* from Macedonia (*Mat. Med*. V.89)); for some, the same name is used for *both* the natural mineral as well as the synthesized one *(kyanos*, *Mat. Med*. V.91); some are synthesized pyrometallurgically (*lithargyros, Mat. Med*. V.87); for others, also artificially produced, a mere cooking hearth would have sufficed (*molybdos kekavmenos* (*Mat. Med.* V.82)), and for yet a third category no heat was required (*psimythion* (*Mat. Med*. V.88)). When they do occur as part of pharmacological recipes, it is not clear whether the natural or the synthesized version has been used; it is surmised that the latter would be a ‘purer’ form of the former.

Therefore, for an assessment of these materials as potential pharmacological agents, it was necessary to break away from the ancient texts and treat G-R MMs as archaeological materials, i.e. like metals, ceramics, glass or metallurgical waste which can be analysed and tested. Given their rarity in the archaeological record our approach involved locating evidence of *similar* minerals ‘deposits’ in the field, within the geographical regions of their purported origin; also locating the archaeological evidence (pottery, installations) confirming that these ‘deposits’ having been worked in the periods of interest (Classical/Hellenistic Roman). Original ‘deposits’ of G-R medicinal minerals are difficult to locate, first because they were removed via quarrying (rather than mining) and second, contrary to, for example, pyrometallurgical waste, the waste products of their enrichment/treatment can be mineralogically/chemically near-identical to the source material.

In the course of the last twenty years, we have been pursuing a geoarchaeological approach in locating minerals/mineral combinations appearing in the G-R texts, in various islands in the Aegean (Photos-Jones and Hall 2011); the red ochre *miltos* in the island of Kea (Photos-Jones et al. 1997, 2018), *Lemnian Earth* in Lemnos (Hall and Photos-Jones 2008; Photos-Jones et al. 2017a), *Samian Earth* in Samos (Photos-Jones et al. 2015), *Melian Earth* and alum group minerals in Melos (Hall et al. 2003a, b; Photos-Jones and Hall 2014); *alumen* in Italy, at Campi Flegrei, near Naples (Photos-Jones et al. 2016) and the Aeolian islands (Photos-Jones et al. 2017b).

However, gradually and in the process of investigating alum extraction and processing on Melos in the localities worked during the Roman period, aspects of the ecology of these landscapes started to come sharply into focus. We became aware that a whole host of microorganisms (readily visible fungi or blue green algae) had made their home in the vicinity of alum group minerals. Nowhere was this more evident than within or around hydrothermal vents (fumaroles) amidst the sparse solfataras of the SE of the island (Photos-Jones et al. 2016, Fig. 4d). Once alerted to their presence, we began to be interested in the alum group minerals’ microbiota (bacteria, fungi, algae, archaea), whether visible or not, and in the need to explore them further. Exposed minerals are often associated with a microbiome. Microbiome is defined as both the microbiota as well as what has been termed their ‘theatre of activity’ (Berg et al. 2020, Fig. 1). The latter is defined as: the structural elements (for example proteins/peptides, lipids, nucleic acids) of the microbial populations present; their biochemical constituents, secondary metabolites, i.e. organic compounds produced by the microorganisms in response to their environment which are not involved in their growth or reproduction; and thirdly, the environmental conditions prevailing in the ecological niche they find themselves in. So, the microbiome can be perceived as ‘a community, its individual members, plus the ‘contributions’ of their everyday life’.

It is well known that the secondary metabolites of many bacteria and fungi are pharmacologically active (as antibacterial, antifungal, anticancer, antitumour, anti-inflammatory) (Shams ul Hassan et al. 2019). Therefore, in investigating G-R medicinal minerals an insight can be gained into natural minerals and their potential bioactivity. If G-R MMs were purported to be therapeutic could their bioactivity be derived from the mineral? its microbiome? or both?

The relationship between mineral and its microbiome is complex because it requires the elucidation of many parameters: for example, does the mineral act simply as a substrate? Or is it used as an energy source for the microorganism? Or is it used by the latter to produce new minerals? (Gross 2018). These questions are beyond the scope of this short study. However, it is known, from the texts, that natural medicinal minerals rarely underwent processing which would have resulted in the destruction of their microbiota, for example, through heating at high temperature. It follows that a substantial portion of the original microbiome (for example bioactive secondary metabolites) would have been carried into the ancient pharmacological recipe, with potentially beneficial (or detrimental) effect.

**Fig. 1:**
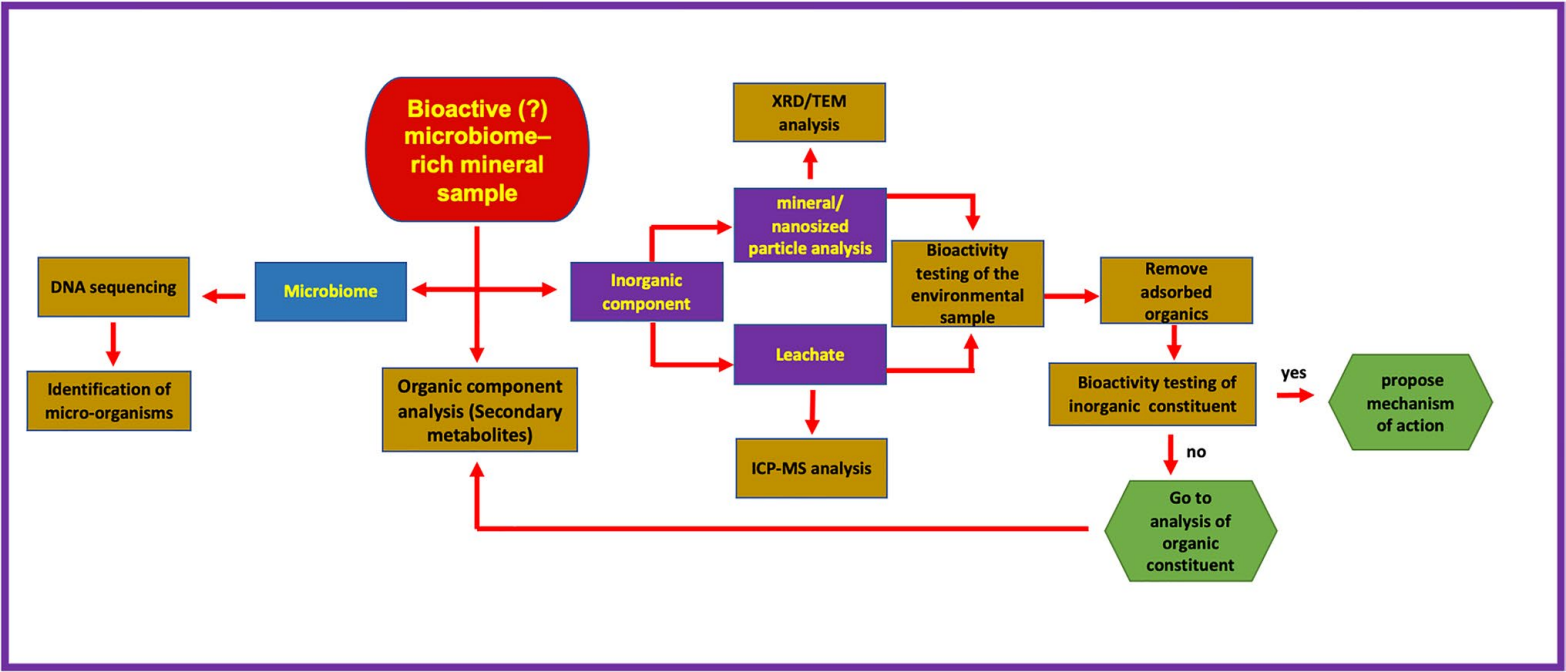
Flowchart of the protocol of work, developed by our team, to test Greco-Roman medicinal minerals as antibacterials, including **a**. the characterization of the organic (microorganisms and their biomolecules) and inorganic (minerals, their nanoparticles and leachates) components and **b**. the bioactivity of each

Given the above, we have devised a protocol of investigation which aimed to a. characterize the two components, the mineral and its biome, independently of each other and b. test the bioactivity of each (Fig. 1) (Photos-Jones et al. 2018; Christidis et al. 2020). Regarding sample characterization, the inorganic component is divided in the powder and the leachate: the former is subjected to mineralogical (at macro level with X-Ray Diffraction and at the nano-scale with Transmission Electron Microscopy) while the latter is subjected to chemical analysis (ICP-MS); the microbial community of the sample is subjected to DNA sequencing to identify the microorganisms present.

Bioactivity testing is focused on the leachate and what is measured is the minimum inhibitory concentration (MIC) of the sample (in mg/mL) that is required to reduce the bacterial colonies by more than 60%. The leachate will carry both inorganic and organic components. Therefore, after measuring the MIC_60_ of the leachate, it is important to separate the organic from the inorganic component in order to test the bioactivity of each separately. The adsorbed organics are removed via oxidation and the (inorganic) leachate is tested again. If the inorganic component is inactive or considerably less bioactive than the original leachate then, it follows, that the organic component (the biomolecules representing secondary metabolites of the original microorganisms) must be the bioactive component; as such it needs to be investigated further via a series of analytical techniques that are best suited for the characterization of the biomolecules within.

**Fig. 2:**
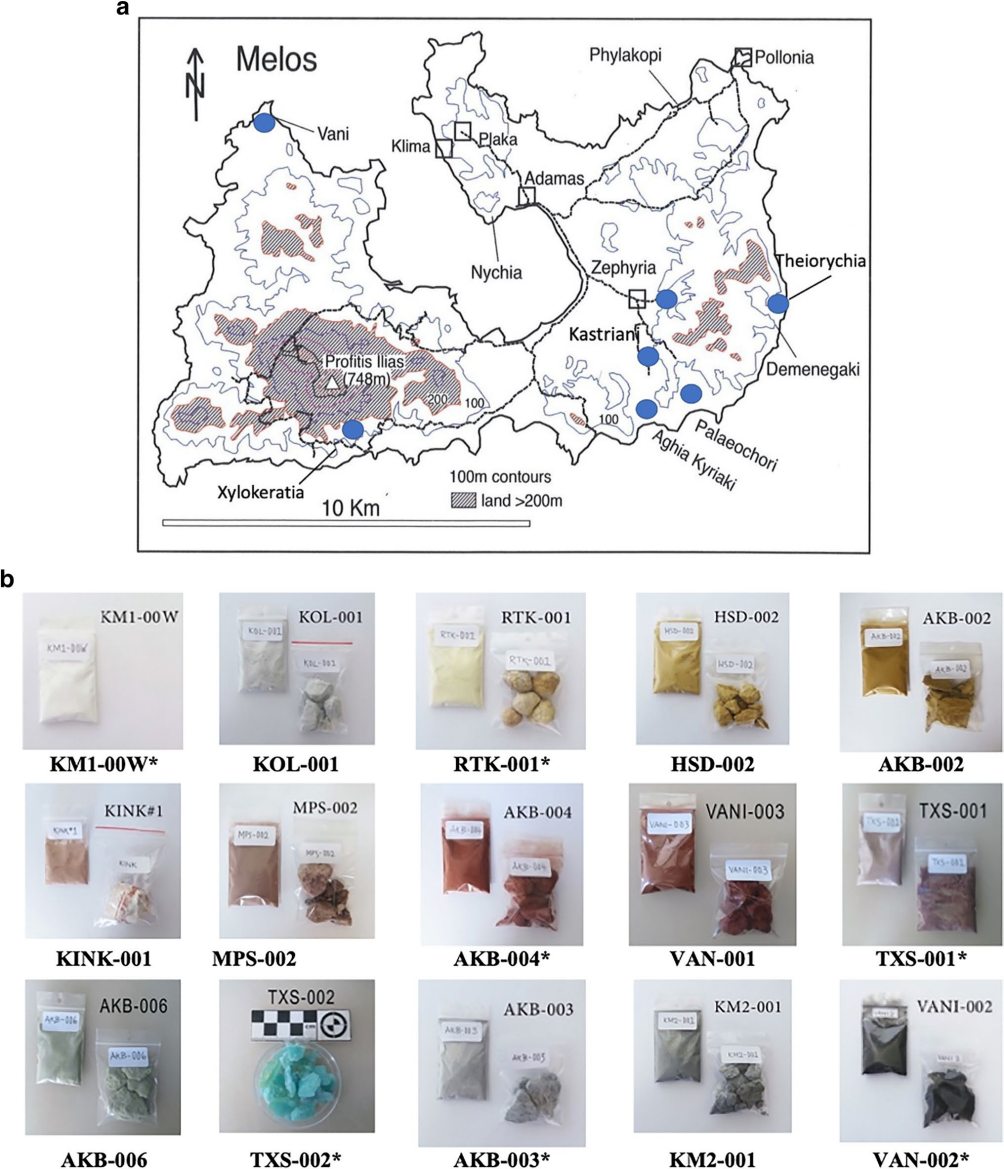
**a** Map of Melos with localities of sampling: ΑΚΒ = Aghia Kyriaki Bay; VAN = Vani; TXS = Theiorychia; MSP/KM = Paleochori bay; HSD = Xylokeratia; RTK = Zephyria; KOL/KINK = Kastriani. Nychia and Demenagaki = obsidian sites; Phylakopi = prehistoric site; Plaka/Adamas /Klima/Pollonia = modern settlements (map adapted from Photos-Jones and Hall 2014). **b** Samples of earth pigments collected from various localities shown in **a**. All samples have been subjected to mineralogical and DNA analyses. Those with a star (*) have also been subjected to antimicrobial testing (MIC_60_)

Having presented our approach, we now turn to earth pigments of various colours. Ochres can vary considerably both in their geological context and in the choice of their use as artist’s pigments. Yellow ochres consist of hydrated iron oxide FeO(OH)·H_2_O, limonite. Red ochres consist of the mineral haematite Fe_2_O_3_ while brown ochres comprise the mineral goethite FeO(OH), (iron oxyhydroxide). Red/yellow ‘ochres’ can also derive from jarosite. Sienna and umber are mixtures of limonite with manganese oxide. ‘Black earths’ can also have an organic origin and result from the burning of wood (charcoal-soot). Green and blue earths are associated with glauconite and celadonite (Delamare and Guineau 2000; Eastaugh et al. 2004; Siddall 2018). The two sections below give a brief reference to the use of ochres in deep time (recent finds in Neanderthal/early modern human sites) and also to the use of red and white in the Greco-Roman world, before focusing attention on samples from the island of Melos, SW Aegean (Fig. 2a).

### About ochres, red miltos and the tetrachromia

Ochres need no introduction to the archaeological record or literature. Specimens of red haematite were found away from source and in association with flint objects at an early Neanderthal site (Maastricht-Belvedere) dated to 200–250 ky in Europe (Roebroeks et al. 2012). In their review of the literature on sites in Africa and Europe with evidence of ochre use, Wolf et al. (2018, 186) write that, particularly from about 140 ky onwards, red/yellow/black earth pigments constitute, in quantity, the third most abundant archaeological find, after lithics and faunal remains. Manganese and iron oxides used in cave paintings in Spain, now dated to 65 ky (Hoffmann et al. 2018) were probably worked by Neanderthals and not modern humans as previously thought. In Africa (Blombos Cave, South Africa) there is now evidence that early modern humans produced graphic designs on hardened mineral crust (silcrete) using a ‘crayon’ of red ochre (haematite) as early at 73 ky ago (Henshilwood et al. 2018). This find predates by many thousands of years the cave art known from the Altamira (Spain) (c. 35–16 ky) and Lascaux (France) (c. 17 ky) caves. Cave art is based on the extensive use of metallic pigments, i.e. iron and manganese oxides for reds, yellows, browns and blacks and with whites provided by minerals like calcite and kaolinite (https://edu.rsc.org/resources/analysis-of-cave-paintings/1586.article).

Beyond the realm of their use as pigments for body decoration and in art, ochres have had many other applications: as tanning agents (Dubreuil and Grosman 2009; Rifkin 2011), mosquito repellents (Rifkin 2015), photoprotective agents against the sun’s UV radiation (Rifkin et al. 2015). Furthermore, their use as healing agents amongst hunter-gatherer groups has found advocates in J Velo (1986a, b); Velo relied on information provided by Peile (1979) on their traditional use by the Aborigines of Australia to cover wounds and to treat burns. Regarding their other uses in deep time (Neanderthals sites) some researchers have argued that ‘manganese dioxide could also function as a combustive agent to produce fire on demand’ (Heyes et al. 2016, in Wolf et al. 2018, 188). The above brief review merely aims to highlight the earth pigments’ great antiquity and their wide range of applications in that time frame.

Coming to the Greco-Roman world, three particular ochres are noted by Dioscorides (*Materia Medica* Book V) for both their medicinal applications and as pigments. Yellow ochre (*Mat. Med.* V.93), was ‘*astringent, septic, dispersive of inflammations and growths*’; White Melian earth (*Mat. Med.* V.159) could *‘cleanse the body and give it a nice color, thin the hair, and cleanse dull-white leprosies*’. Finally, red *miltos* (*Mat. Med* V.96) had ‘*astringent, desiccative, and adhesive properties on account of which it is compounded with plasters for wounds and with troches that dry and make costive’* (English translations, Beck 2005). These three pigments formed part of the *tetrachromia* (four-colour palette), the fourth colour being black. They were particularly favoured by the most illustrious painters of the Classical/Hellenistic and Roman periods. In his well-known passage, Pliny (*Natural History* Book 35, 50) declared that ‘*with only four colours, from the whites, Melian earth, from the yellows, ochre of Attica, from the reds Sinopic miltos and from the blacks atramentum did the illustrious painters Apellis, Aetion, Melathios, and Nichomachos produce their most immortal works*’.

*Miltos,* the red ochre of the *tetrachromia,* had two acclaimed sources*,* Cappadocia, in central Turkey (Pliny *Natural History* Book 35, 30) and the island of Kea (Theophrastus *On Stones*, 52). In the fourth century BCE, it was the latter that was the major producer of this mineral. Our team’s work on Kea *miltos* has shown that it consisted of fine goethite and haematite, quartz, calcite and clay minerals (muscovite, illite, kaolinite) (Photos-Jones et al. 1997, Table 1; 2018). It was known for delivering a striking permanent red. Contemporary authors reported how Athenians, who attempted to abscond from their civic duties, were ‘named and shamed’ by being rounded off in the Agora with a rope dipped in *miltos (*Aristophanes *Ecclesiazusae* 379–380).

But *miltos* was more than a highly staining pigment. It had *other* properties/applications: i.e. as an antifouling agent, and in agriculture as both a fertilizer and as pest control (Lytle 2013). We demonstrated that Kea *miltos’* high content of Pb, Cu, Cd as well as a host of other metallic elements would have been toxic to marine life, therefore most effective as antifouling agent (Photos-Jones et al. 2018, Table 2). We have also shown, through sampling and analyses, that Kea *miltos* carried a host of micro-organisms which are able to convert atmospheric nitrogen to that absorbable by plant roots; this property alone of *miltos*’ natural microbiome would have made *miltos* a good fertilizer.

Critical to our understanding of how *miltos ‘*worked’ is the understanding of the choice of medium in which it was dispensed. For *miltos* to be effective as an antifouling agent, the powder would have had to be mixed with an organic medium, as indeed fourth century BCE inscriptions from Athens (regarding the maintenance of civic walls) make clear when referring to the compound *miltopitta* (or *miltopissa) (miltos*-pitch/tar) (Lytle 2013, 537). For *miltos* to be effective as a fertilizer it would have to be *‘*dissolved’ in water (a leachate of the original powder).

**Fig. 3:**
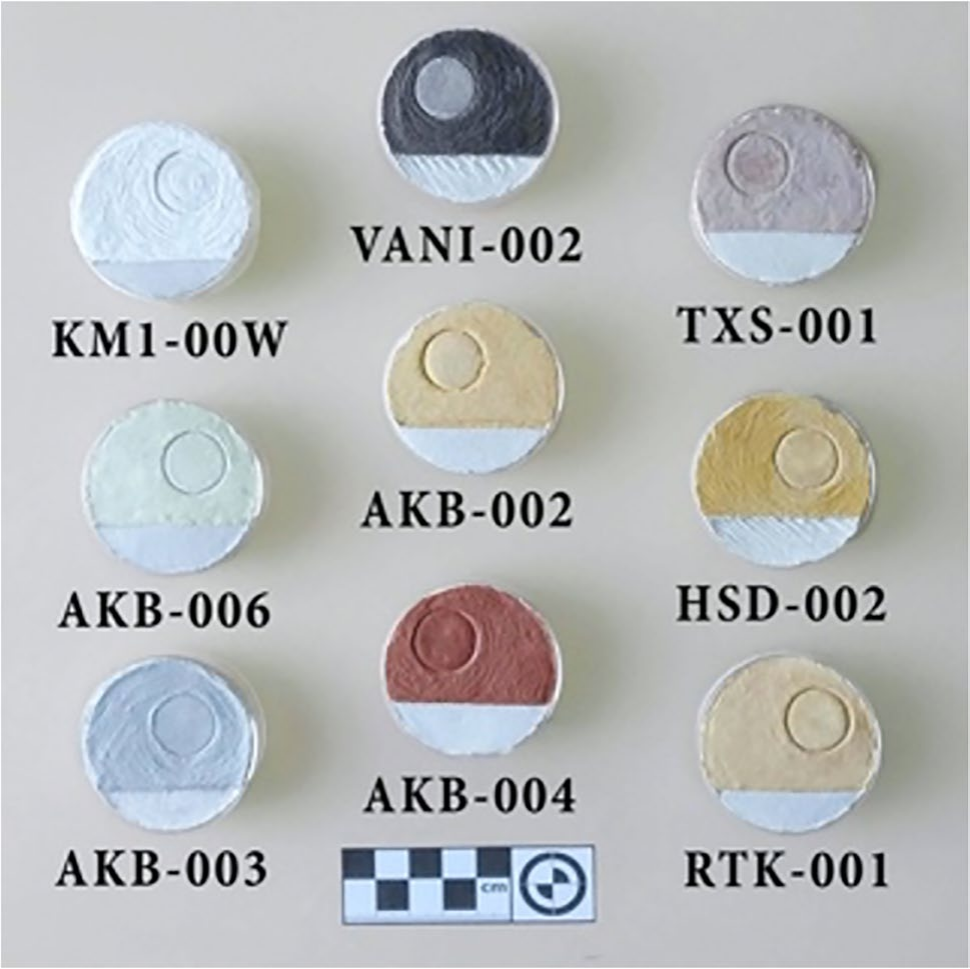
Preparation of some of the samples as pigments for frescoes (see Fig. 2b and Appendix 2). VANI=VAN-002

The focus of this study is red/yellow white, green and black earth pigments from the island of Melos, SW Aegean (Fig. 2a and b). The samples were surface collected by one of us (JGV), a local artist, and from different localities around the island; they were chosen for their suitability for use on plaster. Figure 3 illustrates JGV’s work on plaster using some of the above pigments, while Appendices 1 and 2 provide an outline of the method of preparation and final application in the manner of *buon fresco*. In ‘opting’ to have a local artist guiding the present investigation, this enquiry aimed to take the Melos earth pigments at face value, i.e. as suitable materials for an artist’s palette. Given that the texts ascribe medicinal properties to earth pigments, the question being asked here is: are any *of these* Melian pigments bioactive, i.e. antibacterial?

### Melos geology: A brief background

Melos is situated in the SW part of the South Aegean Volcanic Arc. The geological framework of the island consists of four main units (Fytikas et al. 1986): (i) The Alpine metamorphic basement is characterized by a Middle Eocene high pressure/low temperature metamorphic event overprinted by a high temperature/low pressure one at the boundary between Oligocene and Miocene (Kornprobst et al. 1979; Hoffmann and Keller 1979); (ii) The Neogene marine sedimentary sequence of Upper Miocene age (Fytikas et al. 1986); (iii) A volcanic sequence, which started in the Lower-Middle Pliocene (Fytikas et al. 1986; Stewart and McPhie 2006) and produced both pyroclastics and lavas, of rhyolite to low-Si andesite composition (Fytikas et al. 1986; Stewart and McPhie 2006). The volcanic activity, which occurred under both subaerial and submarine conditions, was initiated in the western part of the island and migrated to the eastern part in the Upper Pliocene-Lower Quaternary (Fytikas et al. 1986; Stewart and McPhie 2006). Subsequent hydrothermal alteration of these volcanics gave rise to extensive bentonite deposits in the eastern part and small kaolin deposits mainly in the western part and the SE of the island as well as a series of shallow marine epithermal deposits in western Melos (Alfieris et al. 2013; Hein et al. 2013; Ivarsson et al. 2019). The fifteen samples shown in Fig. 2b were collected from such areas of hydrothermal activity. Finally (iv) there are alluvial deposits, the most recent in the geological sequence.

## Method

### Samples

**Fig. 4:**
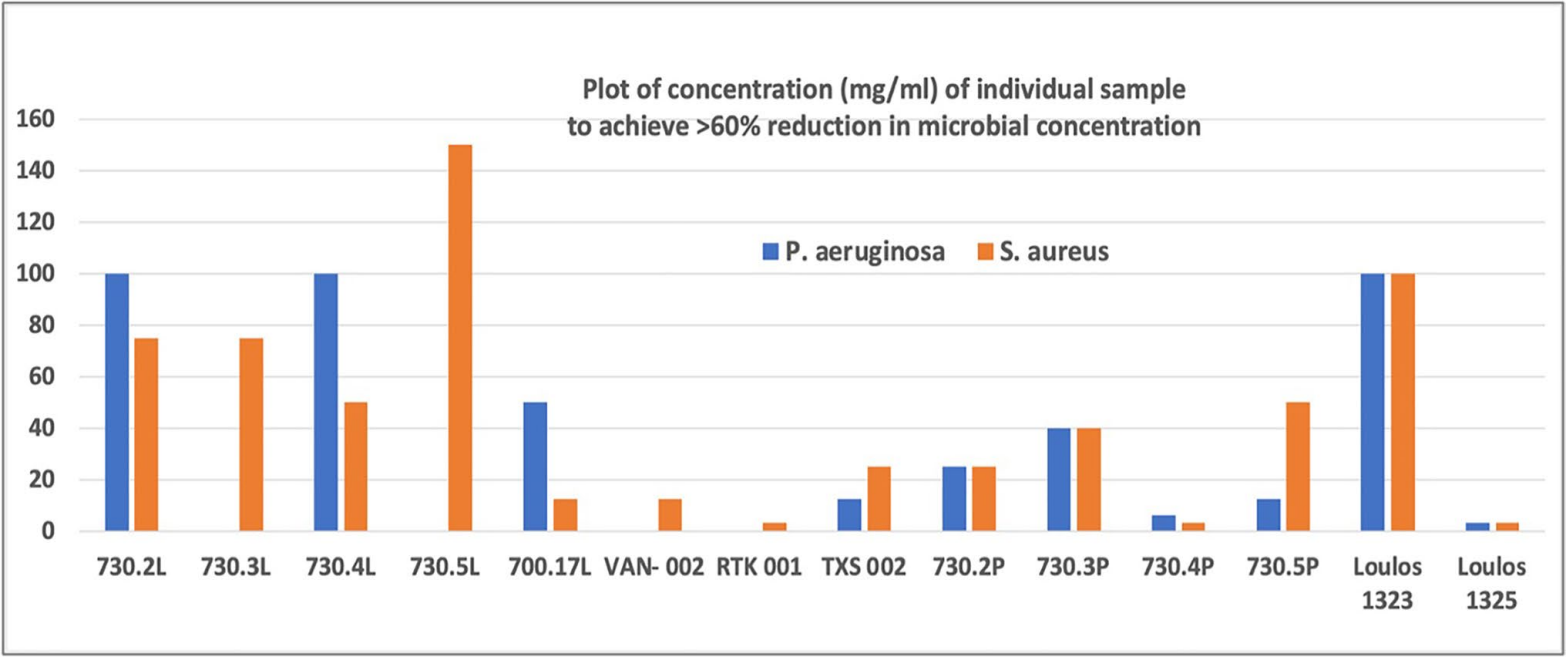
Plot of MIC_60._ for Melos earth pigments and other samples discussed in the text. The smaller the MIC_60_ number, the higher the bioactivity of the said sample

In this paper fifteen Melos pigment-samples (Fig. 2b) were chosen for analysis. For purposes of comparison, we have also included: a. two, previously unpublished, non-pigment samples, also from Melos (Loulos 1323 and Loulos 1325). Loulos is a locality name to the N of Aghia Kyriaki Bay (Fig. 2a). The Loulos samples would not have been considered suitable as pigments on account of their high solubility. They are included here purely for purposes of comparison, one of them being highly bioactive (antibacterial). b. four, previously published, samples of Kea *miltos* (730.2-730.5). Of a total of twenty-one samples, twenty-one have been subjected to XRD analysis (Table 1); three to ICP-OES analysis (Table 2); sixteen to DNA analysis (Table 3); and seven samples to MIC_60_ testing, of which only three were shown to be bioactive (Fig. 4).

### XRD analysis

The mineralogical composition of all samples was determined with X-ray diffraction (XRD), at the School of Mineral Resources Engineering, Technical University of Crete, on a Bruker D8 Advance Diffractometer equipped with a Lynx Eye strip silicon detector, using Ni-filtered CuKα radiation (35 kV, 35 mA). Data were collected in the 2θ range 3–70° 2θ with a step size of 0.02° and counting time 1 s per strip step (total time 63.6 s per step). The XRD traces were analysed and interpreted with the Diffrac Plus software package from Bruker and the Powder Diffraction Files (PDF). The quantitative analysis was performed on random powder samples using Al-holders (side loading mounting approach), by the Rietveld method using the BMGN code (Autoquan© software package version 2.8).

### ICP-OES analysis of leachates

The leachates of three bioactive samples were analysed by Inductively Coupled Plasma-Optical Emission Spectroscopy (ICP-OES) at the Mediterranean Agronomical Institute of Chania (MAICh) using an Agilent 5100 ICP-optical emission spectrometer. The detection limit of the method for analysed elements is less than 1 ppb. The precision of the analyses was tested using elemental standards for all major and trace elements (1000 mg/L) provided by Merck. The relative standard deviation of the analyses varied according to the concentration, typically 6% for the major elements, less for the trace elements.

### Organic carbon analyses

Soil organic matter content as determined by loss of ignition (Hoogsteen et al. 2015). Pre-weighted samples were initially dried at 100 °C overnight, weighed (±0.1 mg), and then heated at 550 C for 3 h (Nabotherm furnace). Correction procedure (Hoogsteen et al. 2015) were applied for structural water loss. Sample VAN-002 (5.0 mg, duplicate), subsequently discovered to have had significant carbon content, was analysed in duplicate by pyrolysis coupled to a two-dimensional chromatography with time-of-flight mass spectrometer, Py-GCxGC-ToFMS, at the University of Strathclyde (Glasgow, UK). A CDS Pyroprobe 6200 to 750 °C heated at a rate of 25 °C/ms connected directly into the Agilent 7890A gas chromatography equipped with a LECO thermal modulator. The inlet (310 °C) was set to a split ratio of 75 with a helium flow rate of 1.4 mL/min. The column set up was reverse phase, 1st dimension column Rxi-17Sil MS (60 m × 0.25 mm i.d. × 0.25 μm; Restek) polar phase, 2nd dimension column less polar phase Rxi-5Sil MS (2.0 m × 0.25 mm i.d. × 0.25 μm; Restek) less polar phase. The primary oven temperature programs were: initial at 50 °C, hold for 0.2 min, ramp 3.5 °C/min to 320 °C, hold for 20 min. The secondary oven and thermal modulator had an offset of +10 °C and + 20 °C respectively from the primary oven temperature. The thermal modulator period was 5 s, and the transfer line temperature was 300 °C. The detector was a LECO (St. Joseph, Michigan, USA) time of flight mass spectrometer (Pegasus 4D) with a spectra acquisition rate of 100 spectra/s. Different compound groups were identified by comparing pyrolusite’s mass spectra to the NIST database (Linstrom and Mallard 2018).

### DNA analysis

DNA extracted with Qiagen DNeasy PowerSoil extraction kit (with pre-incubation at 60 °C for 10 min). Similar handling and quality control measures were followed as according to Christidis et al. (2020). Each 20 μL PCR reaction mixture consisted of 2 μL of DNA sample, 5 μL ssoFast Evagreen reagent (Bio-Rad), 1 μL 10x-primer mixture (0.2 μM final concentration of each primer), and 2 μL molecular-grade water. Reaction conditions were as follows, on a BioRad iCycler5 (BioRad, Hercules, CA USA) instrument: 3-min initial denaturation (94 °C); 40 cycles of: denaturation (10 s at 94 °C), primer annealing (30 s at 58 °C). When completed, the instrument maintained the samples at 8 °C. A post-analytical, high-resolution temperature-melt curve (Δ± 0.05 °C/s). Primers for ‘total bacteria’ (based on 16S rRNA gene) were forward (AYTGGGYDTAAAGNG; position 563–577) and combined set of reverse (TACNVGGGTATCTAATCC, TACCRGGGTHTCTAATCC, TACCAGAGTATCTAATTC, and CTACDSRGGTMTCTAATC; position 907–924). Primers and PCR conditions targeting the 18S-rRNA gene of fungus were based on those by Hadziavdic et al. (2014).

### Antibacterial testing

Antibacterial properties of the samples were tested using two reference bacterial indicators, namely the Gram-negative *Pseudomonas aeruginosa* NCTC 10662 and the Gram-positive *Staphylococcus aureus* NCTC 12493. Both bacteria are often used as indicators in environmental studies and have been reported for their notable adaptability in stressed conditions and importance for public health issues.

The media used for their culture and growth were LB agar (LABM) and LB broth (LABM). Aqueous leachates of the samples were prepared with sterile deionized water at a concentration of 50 mg/mL. Subsequently, ultrasonication was performed in an ultrasonic bath (Julabo) for 30 min at 25 °C, followed by centrifugation at 10,000 g for 15 min for the removal of all solids from the solution. The leachate was decanted and tested against the selected bacterial species for any growth inhibition.

Antimicrobial activity was assessed by means of the broth microdilution method and estimating the Minimum Inhibitory Concentration that inactivated 60% of the bacterial population (MIC_60_). MIC is lowest concentration of an agent that will inhibit the visible growth of a microorganism after incubation. MICs were estimated labelling 96-well sterile microtiter trays with dilutions of each sample. The tested concentration range was 25-0.2 mg/mL. Leachates were inoculated with liquid cultures and bacterial population was adjusted to 10^5^ CFU/mL. The bioactivity of each sample was estimated in relation to positive control wells, which contained only the bacterial inoculum in LB broth. Microtiter trays were incubated at 37 °C for 18–24 h, followed by optical density measurement at 630 nm, using a microplate reader (Labtech LT-4000 Plate Reader) and Manta LML software.

## Results

### XRD — Table 1

Table 1 gives the compositions of the pigments and the minerals’ formulae. As expected, mineralogy determines the colour of the samples. Those samples with white colour (KM1-00 W and KOL-001) are free of Fe-oxides, Fe-oxyhydroxides and/or Mn-oxides and Mn-oxyhydroxides. The yellow colour of samples AKB-002 and HDS-002 is attributed to minor goethite, whereas the red samples VAN-001, AKB-004 and TXS-001 contain haematite with/without goethite. The pale green colour of sample AKB-006 is attributed to the presence of abundant smectite and minor talc. Samples with grey colours contain abundant alunite, whereas the black colour of VAN-002 is due to the presence of abundant hydrous Mn-oxides, namely hollandite and todorokite. Finally, the blue green of sample TXS-002 is due to melanterite (FeSO_4_.7H_2_O). Table 1 also contains the mineralogical composition of *miltos* samples 730.2, 730.3, 730.4 and 730.5 (Photos-Jones et al. 2018) and two samples, from the locality of Loulos, SE Melos, in the proximity of a fumarole (gas emitting vent). As previously mentioned, the Loulos samples do not classify as earth pigments, being highly soluble on account of alunogen and pickeringite.

**Table 1:**
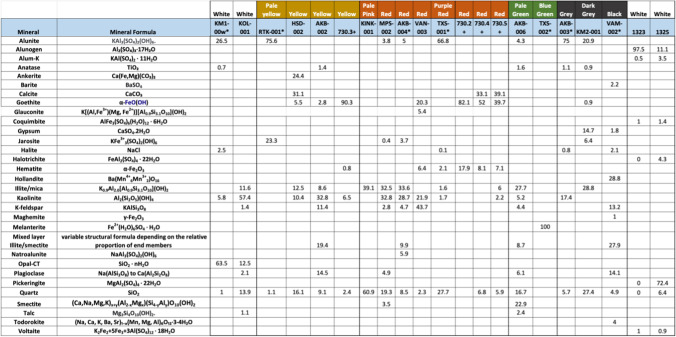
Quantitative XRD analysis (%) of Melos earth pigments; also four *miltos* pigments from Kea (730.2, 730.3, 730.4, 730.5) and two white alunogen/K-alum samples from Melos (LOU-1323 and LOU-1325). Samples marked with a * have been tested for bioactivity

In comparing the Kea *miltos* samples with their red Melos equivalents it is noted that while the former are near-pure iron oxides, the latter would classify as clay-based iron oxides; this is on account of the enhanced levels of kaolinite. In other words, had they not been collected as pigments, by an artist, some of them may have been considered as simply red ‘clays’.

### Analysis of leachates of the bioactive samples — Table 2

Table 2 shows the results of the chemical analysis of the leachates of the three bioactive samples (see “Microbiology testing***”*** section below). The leachates of RTK-001 and VAN-002 are essentially free of heavy metals (Cu, Co, Ni, Zn). Sample VAN-002 contains abundant Na and minor Ca, K, Mg and Mn. By contrast, the leachate of TXS-002 is very rich in Fe and Cu and contains minor amounts of Cu, Co, Ni, Mn and Zn. The source of these elements must have been melanterite, a highly soluble mineral and the only one present in this sample. Bioactivity can relate to high concentrations of some metallic elements, hence the analyses presented in Table 2.

**Table 2:** Chemical composition of leachates of the bioactive samples. Concentrations are in ppm (mg/L). nd = not detected. All entries rounded off to nearest whole number; concentrations presented as ‘0’ denote trace amounts

	RTK-001	VAN-002	TXS-002
B	nd	0	nd
Ca	18	12	15
Co	nd	nd	8
Cu	nd	nd	359
Fe	1	1	4758
K	3	11	nd
Mg	0	6	5
Mn	0	6	5
Na	2	206	0
Ni	nd	nd	19
P	0	0	6
Zn	nd	0	13

### Organic carbon and microbiological eDNA analysis — Table 3

In any environment, bacterial populations are controlled by a number of parameters. These include: the toxicity of the substrates they may be growing on; the availability of energy (food) resource and conditions conducive to their growth (pH, O_2_, temperature or moisture); also, their inoculation potential (for example, can new bacteria establish themselves in location). Generally speaking bacterial populations in agricultural soils can be up to 10^9^ bacteria cells in a gram of soil. Table 3 gives an indication of bacterial (and fungal, if appropriate) populations in each sample.

**Table 3:**
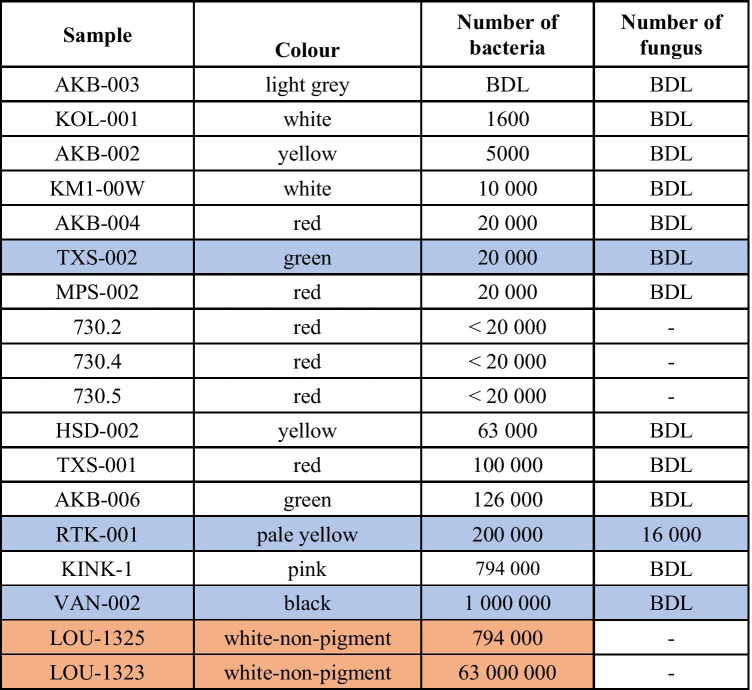
Abundances of bacteria (16S rRNA genes/g soil) and fungus (18S rRNA genes/g soil). *Minimum detection limit = 2000 genes/g. Blue-shaded: samples with high bioactivity; Orange-shaded: Loulos (non-pigment) samples used as ‘controls’

Bacteria concentrations in the earth pigments presented here and measured in genes per gram, are very low. They range from 10^3.3^ (1600) to 10^6.0^ (1 million) genes per gram (Table 3), based on the 16S-rRNA (small subunit ribosome) gene content, a common surrogate measure of the “total bacteria” present (Woese and Fox 1977). The levels detected here are multiple orders of magnitude lower than those normally expected in terrestrial environments which usually range from 10^6^ to 10^9^ cells per gram. Bacteria numbers for samples 730.2, 730.4 and 730.5, discussed in a previous publication (Photos-Jones et al. 2018), are also low, as are bacteria numbers for sample LOU-1325, provided here for comparison. Fungal DNA was only detectable in a single sample, i.e. RTK-001; however, here again levels were slightly above analytical detection limits.

In short, with the exception of VAN-002, all the earth pigments show a considerable paucity of organic content. The only sample with high organic load is LOU-1323. We proceed to investigate closer the nature of the organic load.

Loss of ignition (LOI) was carried out on all samples. With the exception of VAN-002 (LOI = 6%), LOI was found to be minimal (ranging from <1% to 4%). However, once corrected for structural water loss in clay fraction (Hoogsteen et al. 2015), with the exception of VAN-002, values were < 2%, often near 0%. This corresponds with previously reported equally low estimates for LOIs in samples obtained from the region (e.g. Georgoulias and Moustakas 2010).

Pyrolysis (Py-GCxGC-ToFMS analysis) identifies thermally degraded parts of larger organic compounds within samples. Compound identification is carried out by comparing pyrolysis mass spectra to the NIST database (Linstrom and Mallard 2018). With the exception of VAN-002, low concentrations of organics were found in all. Aliphatic compounds, such as branched and n-alkenes, cyclo-alkenes, organic acids and alcohols, are present in all samples, as were mono aromatic compounds (e.g. phenol, toluene, p-xylene, o-xylene). The latter form between 0.6 and 6% of the composition of different samples. We examine here three samples in particular: VAN-002, AKB-002 and AKB-004.

Regarding AKB-002, the main constituent component (46%) is alpha-methylstyrene, most likely a product of plastic breakdown. Other components which contribute a further 42% are acetaldehyde, a commonly occurring compound found in nature and cyclopentane ethyl ethylene. Regarding AKB-004, 76% was made up of N-alkenes, indicating longer chain aliphatic.

Finally, regarding VAN-002, the main pyrolysis products, making up over 65% were diazene, dimethyl- (32.5%) and benzene (32.5%). Both products represent the breakdown of plant materials. Benzene is possibly derived from lignins (Zakzeski et al. 2010; Wu et al. 2017). On the other hand, diazins can represent pyrimidines (e.g., nucleic acids) but also many natural products and biologically active compounds (e.g. Rinderspacher 2014). The remainder pyrolysis components in VAN-002 included alkanes (consisting of 1-heptene (2.5%), 1-octene (2.6)), acetic acid (7.3%), undecanoic acid (5.2%), oxy-acetic acid (3.0%), and cyclohexanone (1.6%). 

### Microbiology testing - Fig. 4

Antibacterial testing was performed on seven samples of the earth pigments (TXS-002, KM-00 W, AKB-004, RT-001, TXS-001, AKB-003 and VAN-002): Fig. 4 shows the MIC_60_ values of only the samples which were found to be bioactive together with those already reported for *miltos* from Kea (Photos-Jones et al. 2018, 730.2–730.5) and also previously unpublished samples for Loulos (1323 and 1325). All MICs are carried out on leachates with the exception of samples 730.2P, 730.4P, 730.5P which were powders.

The results show that only three earth pigments were bioactive against *P. aeruginosa* and *S. aureus*. Specifically, TXS-002, RTK-001 and VAN-002 inhibited bacterial growth with MIC_60_ ranging from 25 to 3.12 mg/mL. The samples VAN-002 (black manganese minerals) and RTK-001 (yellow jarossite) exhibited antibacterial properties only in the case of *S. aureus*, highlighting the different resistance level and behaviour between the two selected bacterial indicators. On the other hand, TXS-002 (melanterite) was active against both species. We point out that the MIC_60_ values of these three earth pigments were much lower (more bioactive) than those measured for the four samples (730.2 L-730.5 L) from Kea and one sample from Loulos (1323). These samples should not, in principle, be considered bioactive, taking into account that inhibitory concentrations for 60% of bacterial reduction were 50–300 mg/mL. Values comparable with the three earth pigments derived only from 730.4P (a powder) and Loulos 1325 (a leachate).

## Discussion

Establishing whether an earth pigment may or may not be bioactive (as antibacterial/antifungal/antioxidant) is a complex question requiring an understanding of: a. the nature (mineralogical/chemical) and ecology of the particular mineral combination; b. the nature of its carrier (aqueous vs. organic). Each sample is examined on its own merit and, so far, there are no overriding criteria as to why some earth pigments, of one or more colours, are antibacterial.

Τhe method we have developed builds up a ‘picture’ of each sample under investigation by profiling its inorganic (mineralogical/geochemical) and organic (microbiome/organic molecules characterization) components. We also test the bioactivity of the leachate (water mixed with powdered sample and subsequently separated from it). Only three samples (black VAN-002, green TXS-002 and pale-yellow RTK-001) displayed considerable antibacterial activity. On the other hand, red, jarosite-containing AKB-004, white, alunite-containing KM1-00 W and AKB-003, and haematite-containing TXS-001 displayed no bioactivity. Similarly low bioactivity was detected in the leachates of the yellow/red Kea miltos 730.2 L, 730.3 L, 730.4 L with 730.5 L being non-bioactive. Contrary to their leachates, the powdered samples 730.2P, 730.3P, 730.4P, 730.5P were bioactive.

Figure 4 combines the results for three samples (VAN-002, TXS-002, RTK-001) with those of previous work on leachates of *miltos* (730.2, 730.3, 730.4, 730.5); the latter containing nanoparticles (average size <200 nm) of Fe-oxides/oxyhydroxides, with minor quartz and mica, have shown limited bacteriostatic activity. On the other hand, the same samples, as powders, showed better bacteriostatic activity, with MIC_60_ values from 3.12 mg/mL upwards (Photos-Jones et al. 2018).

Sample TXS-002 consists of melanterite, an Fe^2+^-bearing hydrous sulphate. During preparation of the leachate, melanterite, which is soluble in water, will release Fe^2+^ and SO_4_^2−^ ions. It is expected that the presence of free Fe^2+^ ions in the leachate will induce antibacterial activity via a Fenton-type oxidation reaction, i.e. reaction of Fe^+2^ with H_2_O_2_, resulting in the generation of free radicals (reactive oxygen species- ROS) which are detrimental to bacteria. This is because Fenton reactions cause oxidative stress on outer cell wall components and this stress can be expanded towards the cytoplasmic membrane, altering cell permeability and leading to the leakage of intracellular components (Cagnasso et al. 2010; Morrison et al. 2016; Williams 2017).

The bioactivity of TXS-002, against both pathogens tested here, verifies the substantial oxidizing power of ROS in effectively reducing microbial populations. *S. aureus* was slightly more resistant; as a Gram-positive bacterium, possessing a thick cell wall, it indicates the higher resistance of that bacterial group under certain environmental conditions (Venieri and Mantzavinos 2017). Additionally, dissolution of melanterite during preparation of leachate released high amounts of Cu and minor amounts of Co, Ni, Zn and Mn, all hosted in melanterite. Copper and Zn^2+^ ions have widely been reported as having antibacterial properties (e.g. Otto and Haydel 2013). In our study, the release of large amounts of Cu^2+^ in the leachate of TXS-002 (Table 2) is bound to affect the antibacterial properties of this sample.

The leachate of RTK-001 also displayed bioactivity (Table 3, Fig. 4). The composition of this sample includes minerals, such as alunite, jarosite and quartz, with very low solubility in water. Therefore, the bioactivity of this leachate cannot be explained in terms of ROS, as in the case of TXS-002. RTK-001 is the only sample with a fungal load as well as a small bacterial load, albeit just above the level of detection. It is not clear whether this combined (bacteria+fungi) organic load is responsible for the bioactivity displayed by RTK-001.

VAN-002 has, again, a different mineralogical composition to the above two earth pigments. It contains Fe-oxides and Fe-oxyhydroxides (haematite and goethite) which are also not soluble. In addition, it is free of Fe^2+^-bearing phases but contains abundant Mn-phases (hollandite and todorokite). This sample also stands apart in having the highest abundance of bacteria (c. 10^6^). These features likely contribute to its considerable antibacterial activity, because they are both expected to be present in the leachate due to their nanoparticle size (Bloise et al. 2020). Todorokite has been shown to have remarkable oxidative properties and to degrade organic compounds, such as dyes. In oxidizing conditions, the active Mn centers of todorokite evolve rapidly through Mn^3+^/Mn^4+^ causing fast catalytic degradation of the dyes (Bletsa et al. 2020). Since todorokite and hollandite contain both Mn^3+^ and Mn^4+^, hollandite is expected to display similar behaviour to todorokite. The presence of minor Mn in the leachate of VAN-002 (Table 2) is in accordance with this suggestion.

Further to the above, VAN-002 contains numerous organic compounds, as described in section 3.2, linked to bacteria populations. The most abundant organic compounds detected in VAN-002 are benzene, diazene, and acetic acid. Benzene rings have been shown to display antibacterial properties (Taleb et al. 2017) and acetic acid has antiseptic properties. Therefore, the organic compounds identified in VAN-002, might also contribute to its antibacterial properties. However, this sample was active only against *S. aureus* and had no effect *on* the *P. aeruginosa* population. This maybe the case because *P. aeruginosa* often exhibits tolerance under stressed conditions, as an opportunistic pathogen (Makropoulou et al. 2018).

We turn now to the overall lack of biomass (with the exception of VAN-002) in the earth pigments examined here and the poor levels of preservation of their bacterial/fungal biome, as demonstrated from pyrolysis analysis. This lack of biomass could be due to the lack of nutrient and resource availabilities needed for the growth of the microbial communities, minimal exposure for inoculation, or environmental toxicities (due to the chemistry of the substrate and/or sunlight /UV radiation exposure (Gutiérrez-Cacciabue et al. 2016; Nelson et al. 2020). Ultraviolet light can not only initiate DNA damage which will affect microbial survival but can also photolytically reduce organic carbon content.

However, although we acknowledge that exposure to UV radiation might have played a role, we note that the *miltos* samples which originated from *within* mines in Kea, also displayed low organic load (Table 3). The latter may or may not be attributed to long storage (samples were collected from these mines more than 20 years ago; Photos-Jones et al. 1997).

TXS-002 and RTK-001 were both bioactive but their bioactivity is likely to be abiotic in nature, i.e. not deriving from secondary metabolite production. VAN-002 was an exception to the above. The black earth pigment had a 6% organic content and a bacterial count of 10^6^ cells/g. VAN-002 had biological activity against *S. aureus,* only. As mentioned, todorokite, one of its constituent minerals, is known for its oxidative properties. It is not clear what the contribution (to bioactivity) of the organic component might be.

## Conclusions

Since the start and throughout our work with G-R medicinal minerals we have assumed that *some,* if not all, *must* have been pharmacologically active. In our attempt to put them to ‘a test’ we begun assessing them for their bioactivity as antibacterials. As our work evolved, we have argued that the origin of that bioactivity is difficult to pinpoint conclusively and can, in principle, derive either from the mineral’s inorganic component or its organic load; or both. Furthermore, that antibacterial activity between powders and leachates of the *same* sample, *can* vary.

Amongst the Melos and Kea earth pigments presented here, amount of organic load varied considerably. We showed that the majority of the red/yellow (Melos and Kea *miltos*) samples did not have a rich bacterial/ fungal load. Melos pale-yellow RTK-001 contained both a small bacterial and a fungal load. But it was the black manganese-rich Melos VAN-002 that was particularly bacteria-rich.

As to which component contributed mostly to bioactivity: in the case of the green pigment TXS-002, it maybe the mineral component thereof (i.e. Fe^+2^ and Cu^+2^ in melanterite) that took a leading role, if it contributed to the generation of ROS via Fenton-type reactions (for Fe^+2^). On the other hand, in the black earth pigment VAN-002, the Mn-rich mineral todorokite, is known to display oxidative powers in degrading organic compounds; but it also appeared not to hinder the growth of bacterial populations. In this case, *both* Mn ions *and* the sample’s microbiome may be driving this particular sample’s bioactivity. From the above, it follows that similarity in colour cannot imply similarity in bioactivity. Yellowish-white RTK-001 is bioactive but equally yellowish-white AKB-002 is not (Fig. 3). 

We conclude that the mechanism(s) driving the bioactivity of the inorganic world (rocks and minerals) and our evolving understanding of it, is far from being elucidated. This is on account of the complex interactions between the organic and inorganic constituent parts at the interface between ecology, mineralogy and bioactivity. Nevertheless, this short paper, albeit preliminary, helps in keeping the discussion ongoing and relevant. We argue that the G-R texts, with their often detailed empirical observations and descriptions of minerals, as pigments, and their inclusions in pharmacological recipes, as therapeutics, can guide and inform this investigation. 

## Appendix 1. by J. Gibney-Vamvakari

The following gives a short account of the treatment of nine of the fifteen samples as shown in Fig. 3. For the preparation of these samples the *buon fresco* (true fresco) technique was used. Each sample was mixed with deionized water and applied onto fresh, wet lime plaster with an artist’s paintbrush and limewater (lime putty diluted with deionized water). *Buon fresco* uses no other binder than the lime in the wet plaster.

- **1. AKB-002**

This sample was found as a soft, solid piece with layers of yellow ochre of different shades and with a strong distinctive smell of soil/rust. It broke up easily into sheets and was ground down easily in a mortar. It needed repeated rounds of levigation to remove the remaining brown colour/impurities. As a fine, non-staining, pigment powder, it seemed to have tiny particles that glistened. It painted onto wet plaster easily as an opaque pigment and dried to a dull ochre yellow.

- **2. AKB-003**

This sample was found as small, compact pieces on sand, most likely having been washed there by rain. It had subtle layers of slightly different colours and crushed easily with a mortar and pestle. It was levigated to remove any sand or debris to become a non-staining opaque paint.

- **3. AKB-004**

This sample was found as a saturated red, compact lump. It was, sieved, ground in a mortar and finally levigated to remove impurities. It was then mulled with water. The final pure pigment was staining and opaque when applied onto plaster. The colour lightened slightly as the plaster dried but still remained strongly saturated.

- **4. AKB-006**

This sample was found combined with sand, and organic impurities. It was processed by levigation; the final, pure pigment was extremely fine so the final test grinding for impurities, in a mortar, was minimal. The pigment was unsaturated and non-staining and was applied easily to wet plaster. The colour lightened as the plaster dried into a non-saturated, dull green.

- **5. KM-00 W**

This sample was found as a fine, crisp white powder that was noticeably brighter in comparison to other ‘white’ samples found on Melos. It was sieved to remove any minute impurities and levigated once to test for the possibility of a ‘brighter’ white pigment. Once the levigation water had completely cleared, no top layer of ‘brighter’ white pigment was distinguishable on the settled white pigment, only a thick top layer that appeared more luminescent, possibly due to its fineness. When removed from the levigation water, the pigment had a lustrous appearance and unique tactile properties, keeping its form when manipulated but reverting to a fine powder when dry. It was ground and mulled before being painted onto the plaster and remained bright as the plaster dried.

- **6. RTK-001**

This sample was found as a solid concretion, initially difficult to break up and grind. It was repeatedly ground in a mortar and sieved to become a fine even-coloured powder that was not levigated as it had no visible impurities. The resulting pigment was bright, opaque and non-staining with a creamy consistency that painted easily onto wet plaster. While drying, the pigment slowly changed from bright yellow to an orange/red colour, possibly reverting back to the reddish tones of jarossite.

- **7. HSD-002**

was found fallen from a small cliff of the same material, as a large solid piece that was soft after rain. It crushed easily with a mortar and pestle into a fine powder, which was not initially levigated as it seemed pure and clean with no obvious impurities such as grit or plant roots. It became a creamy, easy to use opaque pigment, and when levigated separated into a darker and lighter hue with the same properties.

- **8. TXS-001**

This sample was found as a soft, pink powder combined with mineral and organic impurities. It was sieved and levigated many times resulting in a very fine powder; it required minimal grinding. This non-staining pigment was applied easily to wet plaster and lightened slightly when dry.

- **9. VAN-002**

This sample was collected as fist-sized black lumps. It was sieved and ground to become a heavily staining, fine, black powder. It was not levigated. The pigment was applied easily to wet plaster and lightened slightly when dry.

## Appendix 2. by J. Gibney-Vamvakari

### Colour Swatches

Lime plaster was chosen as the substrate for colour swatches. Using the *buon fresco* technique, the prepared pigments, ground in water, were applied to the final smooth, wet plaster layer (the *intonaco*). Over time the pigments merge with the plaster to become an integral part of the surface due to the process of carbonation when the lime putty slowly reverts back to limestone. The lime plaster consisted of an aggregate (river sand) and mature lime putty.

To ensure the river sand was not contaminated with silt or clay, it was meticulously washed in rainwater and repeatedly rinsed in deionized water. This was the most time-consuming part of the prep process. Once the sand was absolutely dry it was sieved with a 40 and 60 mesh sieve.

Two batches of lime plaster were made: Batch 1 was made for the first two layers of plaster by using 2 parts of 40 m sand to 1 part lime putty; Batch 2 was made for the final smooth intonaco layer using 1 part 60 m sand to 0.75 parts lime putty. Both were thoroughly mixed (with **no** extra water) sealed in airtight containers and allowed to rest for at least 24 h before use.

Before each use, the plaster was well kneaded with artist palette knives to make it more pliable. The base of each dish was lined with a disc of hessian fabric previously soaked in deionized water.

#### Layer 1

25 g of plaster from batch 1 was pressed into the base of the dish with artist palette knives and the surface left rough to allow the next layer to adhere/meld easily. It was allowed to dry thoroughly which can take days, depending on weather conditions.

#### Layer 2

Once dry the base layer was brushed to remove any loose particles and well moistened with deionized water. 15 g of plaster from batch 1 was pressed onto the base layer and the surface smoothly and neatly finished. This second layer wasn’t allowed to dry before the next layer was applied.

#### Layer 3

10 g of plaster from batch 2 was added immediately for the final intonaco layer, the surface was carefully and neatly smoothed with artist palette knives and scored with painting guidelines.

Painting onto the intonaco layer started immediately. The colour swatches for this project were made with three layers of lime plaster specifically prepared for small-scale work (using fine grades of sand) and 6 cm plastic petri dishes as containers (see Fig. 3).
